# Dengue Nonstructural Protein 1 Maintains Autophagy through Retarding Caspase-Mediated Cleavage of Beclin-1

**DOI:** 10.3390/ijms21249702

**Published:** 2020-12-19

**Authors:** Zi-Yi Lu, Miao-Huei Cheng, Chia-Yi Yu, Yee-Shin Lin, Trai-Ming Yeh, Chia-Ling Chen, Chien-Chin Chen, Shu-Wen Wan, Chih-Peng Chang

**Affiliations:** 1 Department of Microbiology and Immunology, College of Medicine, National Cheng Kung University, Tainan 701, Taiwan; dora927123@gmail.com (Z.-Y.L.); yslin1@mail.ncku.edu.tw (Y.-S.L.); 2 School of Medicine for International Students, College of Medicine, I-Shou University, Kaohsiung 824, Taiwan; cherish216@gmail.com; 3 National Institute of Infectious Diseases and Vaccinology, National Health Research Institutes, Miaoli 350, Taiwan; epitope@nhri.org.tw; 4 Center of Infectious Disease and Signaling Research, National Cheng Kung University, Tainan 701, Taiwan; today@mail.ncku.edu.tw; 5 Department of Medical Laboratory Science and Biotechnology, College of Medicine, National Cheng Kung University, Tainan 701, Taiwan; 6 School of Respiratory Therapy, College of Medicine, Taipei Medical University, Taipei 110, Taiwan; chialing66@tmu.edu.tw; 7 Pulmonary Research Center, Wan Fang Hospital, Taipei Medical University, Taipei 116, Taiwan; 8 Department of Pathology, Ditmanson Medical Foundation Chia-Yi Christian Hospital, Chiayi 600, Taiwan; hlmarkc@gmail.com; 9 Department of Cosmetic Science, Chia Nan University of Pharmacy and Science, Tainan 717, Taiwan; 10 Department of Medical Laboratory Science, College of Medicine, I-Shou University, Kaohsiung 824, Taiwan; 11 The Institute of Basic Medical Sciences, College of Medicine, National Cheng Kung University, Tainan 701, Taiwan

**Keywords:** dengue virus, autophagy, apoptosis, Beclin-1, caspase, NS1

## Abstract

Dengue virus (DENV) infection is a significant public health threat in tropical and subtropical regions; however, there is no specific antiviral drug. Accumulated studies have revealed that DENV infection induces several cellular responses, including autophagy and apoptosis. The crosstalk between autophagy and apoptosis is associated with the interactions among components of these two pathways, such as apoptotic caspase-mediated cleavage of autophagy-related proteins. Here, we show that DENV-induced autophagy inhibits early cell apoptosis and hence enhances DENV replication. Later, the apoptotic activities are elevated to suppress autophagy through cleavage of Beclin-1, an essential autophagy-related protein. Inhibition of cleavage of Beclin-1 by a pan-caspase inhibitor, Z-VAD, increases both autophagy and viral replication. Regarding the mechanism, we further found that DENV nonstructural protein 1 (NS1) is able to interact with Beclin-1 during DENV infection. The interaction between Beclin-1 and NS1 attenuates Beclin-1 cleavage and facilitates autophagy to prevent cell apoptosis. Our study suggests a novel mechanism whereby NS1 preserves Beclin-1 for maintaining autophagy to antagonize early cell apoptosis; however, elevated caspases trigger apoptosis by degrading Beclin-1 in the late stage of infection. These findings suggest implications for anti-DENV drug design.

## 1. Introduction

Dengue disease is caused by dengue virus (DENV) infection, and it places a heavy socioeconomic and disease burden on many tropical and subtropical regions [1]. DENV is a positive-sense, single-stranded RNA virus. The RNA genome of DENV is translated into a single polyprotein which includes three structural (capsid, premembrane/membrane, envelope) proteins and seven nonstructural (NS1, NS2A, NS2B, NS3, NS4A, NS4B, NS5) proteins [2]. However, there are no licensed antiviral drugs or therapeutics against DENV infection. For the development of antiviral drugs, viral factors and cellular factors, which are required for the replication of DENV, are important targets [3,4]. Virus infection can cause significant changes to the host cells, including cellular metabolism and homeostasis, to assure their own propagation. Studies have shown that DENV manipulates several cellular pathways, such as autophagy, apoptosis and endoplasmic reticulum (ER) stress during their life cycle [2,5,6,7,8]. Autophagy is not only a process of self-eating by degrading a cell’s own components within lysosomes, but it also helps to eliminate invaded viruses in autophagosomes [9]. However, some reports have demonstrated that the autophagic machinery activated by DENV infection is able to enhance its replication [6,10,11,12,13]. In another way, apoptosis is a process of programmed cell death that regulates cellular homeostasis by removing damaged, infected, or excess amounts of cells. Several studies showed that both extrinsic and intrinsic apoptosis could be induced by dengue virion or viral proteins. DENV isolated from clinical samples induced apoptosis in vitro, including endothelial cells [14,15], liver cells [16] and dendritic cells [17,18]. Cell apoptosis can eliminate infectious microbes and serve as an innate immunity mechanism. Accordingly, DENV has been found to develop certain strategies to inhibit apoptosis in the early life cycle to ensure viral replication. For example, activation of the phosphatidylinositol 3 kinase/Akt (PI3K/Akt) pathway maintains cell survival by inhibiting caspase-dependent cell death in the early stages of DENV infection [19]. Our current study further pointed out that the PI3K/Akt pathway and 78-kDa glucose-regulated protein/Binding immunoglobulin protein (GRP78/Bip) are both required for DENV early replication [20].

Autophagy and apoptosis are both essential in regulating the homeostasis of cells. The connection between autophagy and apoptosis is currently observed to be under the control of multiple common upstream signals, which also cross-regulate each other in an inhibitory manner. In general, autophagy blocks the induction of apoptosis and helps cells adjust to environmental stress; however, accumulated external signals may in turn activate apoptosis-associated molecules and shut off the autophagic process [21]. Autophagy can attenuate or inhibit cell apoptosis by reducing the stability or activity of pro-apoptotic proteins. For example, autophagy related 7 (ATG7) could repress caspase-8 or caspase-9 activity to inhibit apoptosis [22,23]. On the other hand, a high degree of caspase activation induced by apoptosis can lead to the cleavage of several essential autophagy proteins to suppress autophagy [21]. In addition, the small fragments of these degraded ATG proteins, including ATG4D, ATG5, and Beclin-1, subsequently enhance apoptosis [24,25]. Although DENV infection is known to induce both autophagy and apoptosis, the cross-regulation between these two processes in DENV-infected cells is still not fully understood.

Beclin-1 serves as a checkpoint between autophagy and apoptosis due to the interaction of the Bcl-2 family protein [26]. Beclin-1 is a 60 kilo-Dalton (kDa) protein which contains three identified structural domains, a Bcl-2 homology domain (BH3) at the N-terminus, a coiled-coil domain (CCD), and an evolutionarily conserved domain (ECD); all three domains enable Beclin-1 to interact with multiple proteins. The autophagic activity of Beclin-1 is regulated by protein–protein interaction (e.g., Bcl-2 or Bip) or post-translational modification (e.g., phosphorylation or ubiquitination) [26,27,28,29,30,31]. Reports have shown that flaviviruses take advantage of autophagy by using ER-derived membranes to facilitate their replication [13]. DENV-induced ER stress increases autophagy activity and viral replication through the inositol-requiring protein-1α (IRE1α)-c-Jun N-terminal kinase (JNK) pathway. The phosphorylated JNK further induces Bcl-2 phosphorylation and causes dissociation of the Beclin-1-Bcl-2 complex, which is able to initiate autophagy and facilitate DENV replication [6,7]. On the other hand, several caspases are involved in the cleavage of Beclin-1, such as caspase-3, 6, 8, 9 and 10, which suppress Beclin-1-mediated autophagy [32,33,34,35,36,37]. In addition, autophagy deregulation occurred during retinal ischemia and was associated with calpain-mediated Beclin-1 cleavage [38]. These studies indicate that the cleavage of Beclin-1 inhibits autophagy to promote apoptosis. However, it remains unclear whether Beclin-1 triggered autophagy prevents DENV-induced cell death.

Other reports have shown that DENV NS1 supports viral replication through interacting with viral replicase proteins (such as NS4A and NS4B) as well as multiple host proteins (such as RNA Helicase A and human heterogeneous nuclear ribonucleoprotein C1/C2) [39,40,41]. These interactions may not only contribute to virus replication but also to cellular responses which are favorable for DENV survival in host cells. Notably, a study has suggested that the colocalization of DENV NS1 and amphisomes, organelles formed by the fusion of autophagosomes with endosomes, indicates an autophagy-associated docking site for viral replication [11]. However, the roles of NS1 in facilitating viral replication through maintaining autophagy need to be further investigated.

In this study, we explored the crosstalk between DENV-induced autophagy and apoptosis. We revealed that DENV-induced autophagy prevents early cell apoptosis and facilities viral replication. DENV NS1 was found to protect Beclin-1 from caspase-mediated cleavage to maintain the autophagy function. However, elevated apoptotic activities suppress autophagy by inducing fragmentation of Beclin-1, which leads to cell apoptosis in the late stages of DENV infection.

## 2. Results

### 2.1. Inhibition of Autophagy Enhances DENV-Induced Apoptotic Cell Death

**Figure 1:**
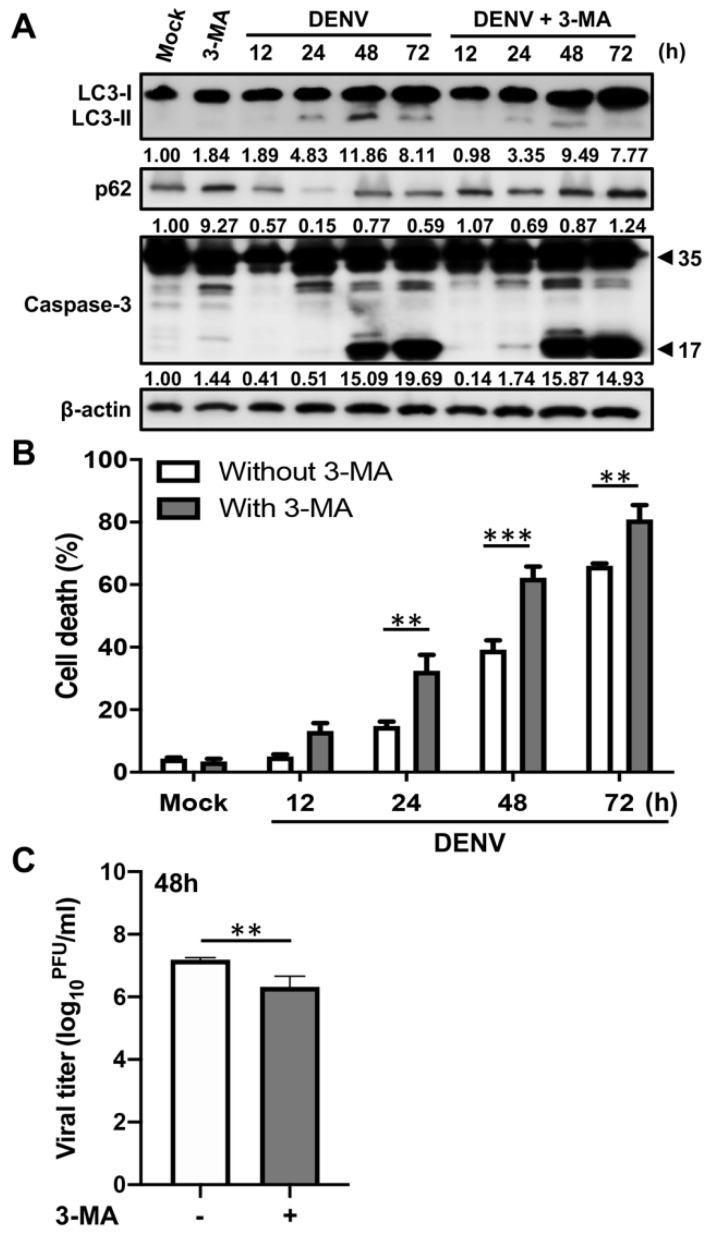
Inhibition of autophagy enhances DENV-induced apoptosis. A549 cells were pretreated with or without 5 mM 3-Methyladenine (3-MA) and then infected with DENV at the multiplicity of infection (MOI) = 2 for the indicated time. (**A**) Western blot analysis showing representative images of LC3-II accumulation, p62 degradation as well as procaspase-3 (35 kDa) and cleaved caspase-3 (17 kDa) levels in A549 cells. β-actin is shown for loading control. The blots were quantified by using β-actin as a calibrated control. The relative ratios (numbers below the blots) of LC3-II, p62 or cleaved caspase-3 to mock were indicated. (**B**) The percentage of cell death was determined by propidium iodide staining. Results shown are means ± SD for triplicate cultures. Statistical significance was based on two-way ANOVA followed by Bonferroni’s multiple comparison test. (**C**) The viral titers were determined by plaque assay. Results shown are means ± SD for triplicate cultures. Statistical analysis was performed using a two-tailed Student *t*-test. All the data are representative of independent experiments. ** *p* < 0.01, and *** *p* < 0.001.

To determine the relationships among autophagy, apoptosis and DENV replication, the A549 cells were infected with DENV in the presence or absence of the autophagy inhibitor, 3-methyladenine (3-MA), for 12 to 72 h. At each timepoint, cells were harvested from 3 concomitant infections, and expressions of the following were determined by Western blot: LC3-II as a surrogate for autophagosome accumulation; p62 as a surrogate for ubiquitinated protein sequestration; and caspase-3 as a marker of apoptosis. Time-course analysis revealed LC3-II accumulation and p62 degradation in the early stages (12–24 h post-infection), but an increase in cleaved caspase-3, an indication of apoptosis, was observed at 48 h post-infection (Figure 1A). Next, we measured cell death by propidium iodide uptake assay and determined viral titers by plaque assay. The increased level of cleaved caspase-3 was associated with elevation of cell death rates (Figure 1B). These data indicated that DENV induces autophagy before apoptosis during infection. While autophagy was inhibited by 3-MA, cleaved caspase-3 was detected as early as 24 h post-infection (Figure 1A). Furthermore, cell death rates were increased within 24 to 72 h post-infection (24 h post-infection: 14.77 ± 2.56% versus 32.47 ± 8.82%, about 2.20-fold change, *p* < 0.01; 48 h post-infection: 39.17 ± 5.20% versus 62.17 ± 6.31%, about 1.59-fold change, *p* < 0.001; 72 h post-infection: 66.07 ± 1.27% versus 80.87 ± 7.96%, about 1.22-fold change, *p* < 0.01), whereas viral titers were decreased by 1.34 logs (*p* < 0.01) at 48 h post-infection in the presence of 3-MA (Figure 1B,C). These results suggest that DENV induces autophagy to inhibit early cell apoptosis and help DENV replication. However, in the late stage of DENV infection, apoptosis activities are elevated to suppress autophagy and hence triggering severe cell death. These findings suggest that there is crosstalk between autophagy and apoptosis during the DENV replication cycle.

### 2.2. Beclin-1 Protects Cells from Early DENV-Induced Apoptosis

**Figure 2:**
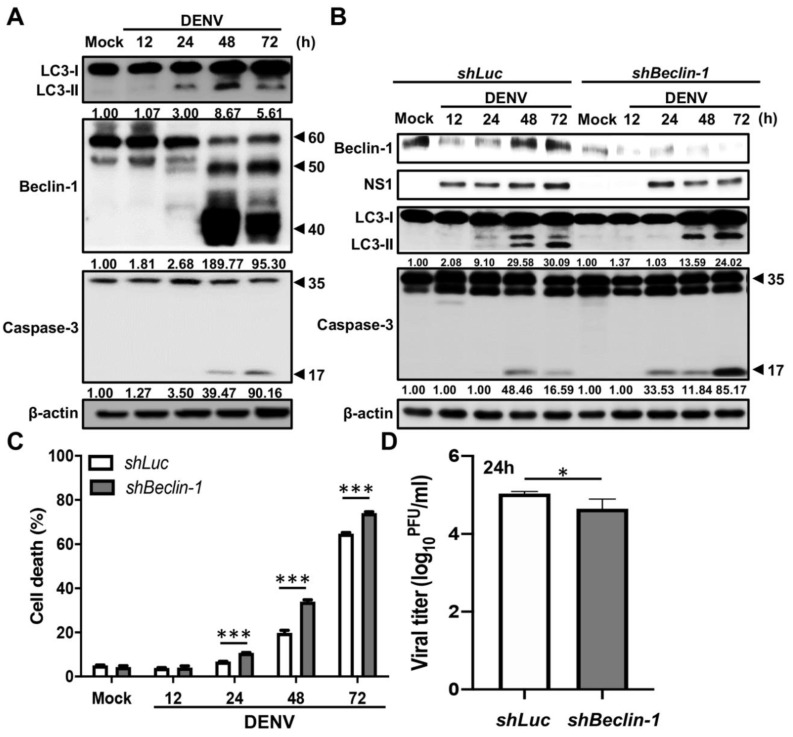
Beclin-1 ablation promotes cell death and inhibits virus production in DENV-infected cells. (**A**) A549 cells were infected with DENV at MOI = 2 for the indicated time. Western blot analysis shows representative images of LC3-I and LC3-II levels, Beclin-1 levels (full-length: 60 kDa, cleaved form: around 50 and 40 kDa), as well as procaspase-3 (35 kDa) and cleaved caspase-3 (17 kDa) levels in mock and DENV-infected cells. β-actin is shown for loading control. The blots were quantified by using β-actin as a calibrated control. The relative ratios (numbers below the blots) of LC3-II, cleaved Beclin-1 (40 kDa) or cleaved caspase-3 to mock were indicated. (**B**) shLuc and shBeclin-1 A549 cells were infected with DENV at MOI = 2 for indicated time. The expression of Beclin-1, NS1, LC3 and caspase-3 was analyzed by Western blotting. β-actin is shown for loading control. The blots were quantified by using β-actin as a calibrated control. The relative ratios (numbers below the blots) of LC3-II or cleaved caspase-3 to mock were indicated. (**C**) The percentage of cell death was determined by propidium iodide staining. Results shown are means ± SD for triplicate cultures. Statistical significance was based on two-way ANOVA followed by Bonferroni’s multiple comparison test. (**D**) The viral titers were determined by plaque assay. Results shown are means ± SD for triplicate cultures. Statistical analysis was performed using a two-tailed Student *t*-test. All the data are representative of independent experiments. * *p* < 0.05, and *** *p* < 0.001.

Accumulated external signals may in turn activate apoptosis-associated molecules and shut off the autophagic process by the degradation of ATG proteins. Several ATG proteins, including ATG5 and Beclin-1, could be cleaved by calpains or caspases that were activated during apoptosis [30,35]. To determine whether the degradation of autophagy related proteins contributes to the regulation between autophagy and apoptosis in DENV-infected cells, we checked several essential autophagic proteins, including ATG5, ATG5-12 complex, ATG7, ATG9, ATG14 and Beclin-1 during DENV infection. The expression levels of ATG5, ATG7, ATG9, and ATG14 were all increased dramatically in the late stage of DENV infection, while the level of ATG5-ATG12 conjugated complex did not notably change (Figure S1). Interestingly, although there is no change of Beclin-1 expression from 12 to 24 h post-infection, a decrease in the full length of Beclin-1 (around 60 kDa) and an increase in cleavage forms (around 50 and 40 kDa) were notable at 48 h post-infection. In addition, the cleavage of Beclin-1 was accompanied by the cleavage of caspase-3, indicating that DENV infection leads to both Beclin-1 degradation and apoptosis at a later phase of the replication cycle (Figure 2A).

To understand the roles of Beclin-1 in DENV-induced autophagy and apoptosis as well as in viral replication, we established Beclin-1 knockdown A549 cells (shBeclin-1) by lentiviral-based RNA interference technology. The Luciferase knockdown (shLuc) A549 cells were also generated as control cells. After DENV infection, we found that the cleaved caspase-3 was increased (Figure 2B), as were cell death rates, within 24 to 72 h post-infection (24 h post-infection: 6.78 ± 0.172% versus 10.74 ± 0.25%, about 1.58-fold change, *p* < 0.001; 48 h post-infection: 19.81 ± 1.94% versus 33.97± 1.42%, about 1.17-fold change, *p* < 0.001; 72 h post-infection: 64.79 ± 0.85% versus 74.07 ± 0.91%, about 1.14-fold change, *p* < 0.001) in Beclin-1 knockdown cells compared with control cells. On the other hand, the LC3-II accumulation decreased within 24 to 72 h post-infection (Figure 2B) and viral tilters were reduced by 0.56 logs (*p* < 0.05) at 24 h post-infection (Figure 2D) in Beclin-1 knockdown cells compared with control cells. These results indicated that Beclin-1 plays an anti-apoptosis role in DENV-infected cells. The lack of full-length Beclin-1 may enhance apoptosis by inhibiting autophagy and hence leads to cell death.

### 2.3. Caspases Mediate the Cleavage of Beclin-1 in DENV-Infected Cells

**Figure 3:**
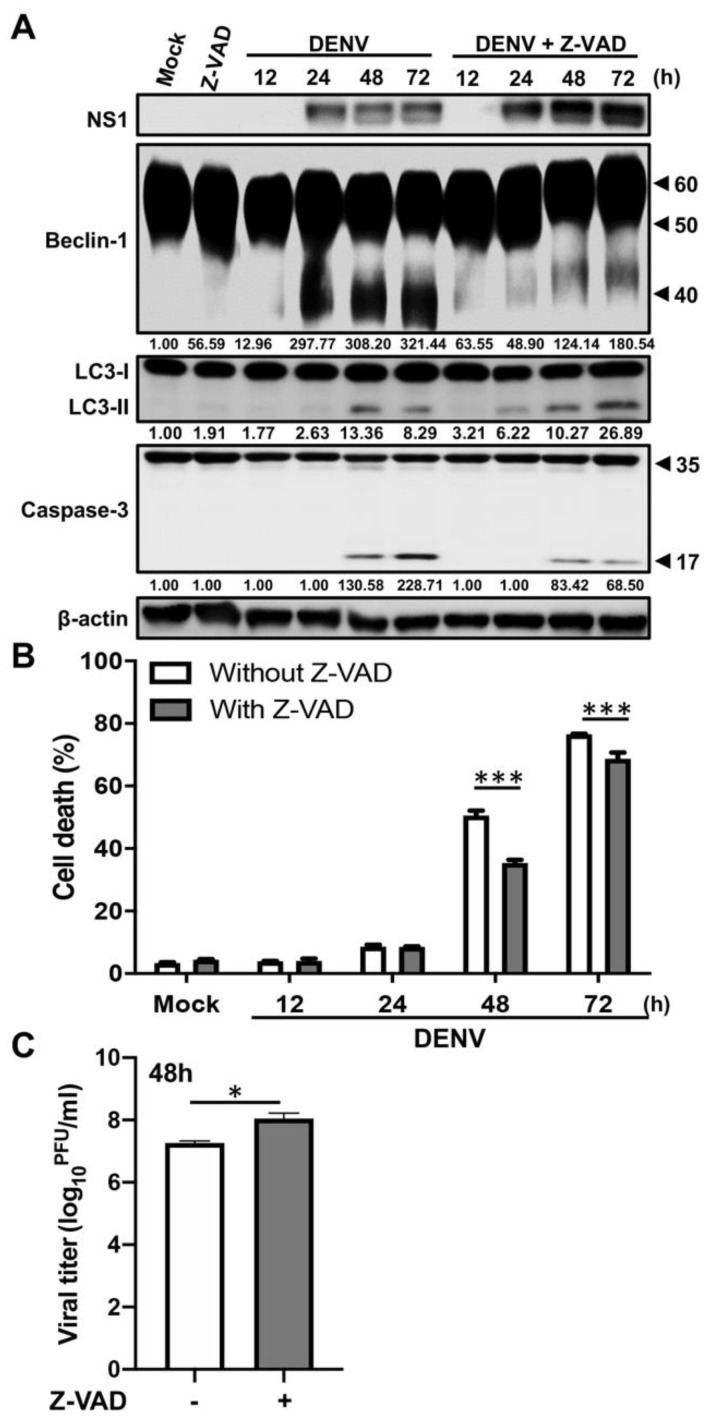
Caspases mediate cleavage of Beclin-1 in the DENV-infected cells. A549 cells were pretreated with or without 25 μM Z-VAD and then infected with DENV at MOI = 2 for the indicated time. (**A**) Western blot analysis showing representative images of NS1 levels, Beclin-1 levels (full-length: 60 kDa, cleaved form: around 50 and 40 kDa), LC3-II accumulation, as well as procaspase-3 (35 kDa) and cleaved caspase-3 (17 kDa) levels in A549 cells. β-actin is shown for loading control. The blots were quantified by using β-actin as a calibrated control. The relative ratios (numbers below the blots) of LC3-II, cleaved Beclin-1 (40 kDa) or cleaved caspase-3 to the mock were indicated. (**B**) The percentage of cell death was determined by propidium iodide staining. Results shown are means ± SD for triplicate cultures. Statistical significance was based on two-way ANOVA followed by Bonferroni’s multiple comparison test. (**C**) The viral titers were determined by plaque assay. Results shown are means ± SD for triplicate cultures. Statistical analysis was performed using a two-tailed Student *t*-test. All the data are representative of independent experiments. * *p* < 0.05, and *** *p* < 0.001.

Previous studies showed that under strong stress, the activated cellular proteases, such as calpains or caspases, are able to degrade Beclin-1 and further trigger apoptosis [30,35]. We next tested whether protease-mediated cleavage of Beclin-1 is involved in the late stage of DENV infection. The inhibitors of calpain and caspase, MDL28710 and Z-VAD, respectively, were used. First, the A549 cells were pre-treated with MDL28710 and then infected with DENV. According to the results shown in Figure S2, there were no differences in the Beclin-1 fragmentation, cell death rate or viral titers between the cells with or without MDL28710 treatment. Hence, this suggests that DENV-induced Beclin-1 cleavage is not mediated by calpain. Next, we determined whether caspases mediate the cleavage of Beclin-1 in DENV-infected cells. Thus, the A549 cells were pre-treated with Z-VAD and infected with DENV, sequentially. Results showed that Z-VAD inhibited DENV-induced caspase-3 cleavage (Figure 3A) and cell death rates (48 h post-infection: 50.50 ± 2.88% versus 35.37 ± 1.78%, about 0.70-fold change, *p* < 0.001; 72 h post-infection: 76.50 ± 0.40% versus 68.73 ± 3.38%, about 0.90-fold change, *p* < 0.001) (Figure 3B). Moreover, inhibition of caspase activity not only reduced Beclin-1 cleavage but also increased LC3-II accumulation (Figure 3A) and viral titers by 0.85 logs (*p* < 0.05) at 48 h post-infection (Figure 3C). These results suggest that DENV-activated caspases not only degrade Beclin-1 but also promote apoptosis. The preservation of full-length Beclin-1 and autophagy might support viral replication during the DENV life cycle.

### 2.4. DENV NS1 Retards Beclin-1 Degradation and Promotes Autophagy to Inhibit Apoptotic Cell Death

**Figure 4:**
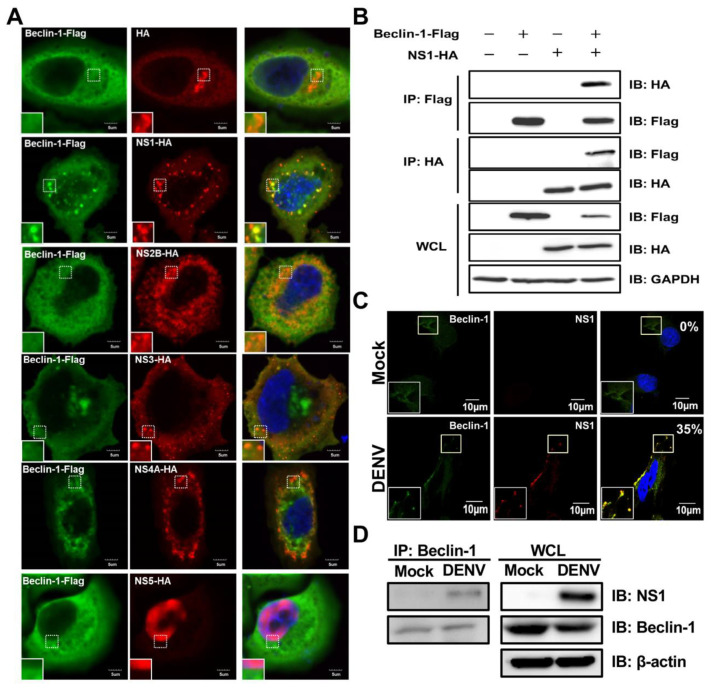
The colocalization and interaction of DENV NS1 with Beclin-1 are found in NS1-expressing and DENV-infected cells. (**A**) Hela cells were co-transfected with NS plasmids (including HA, NS1-HA, NS2B-HA, NS3-HA, NS4A-HA or NS5-HA) and Belcin-1-Flag plasmids for 48 h. At 48 h post-transfection, cells were fixed, permeabilized, and stained with anti-HA Abs (green), anti-Flag Abs (red) and DAPI (blue). Cells were mounted and observed by confocal microscopy. The square insets show higher magnification. Scale bar: 5 μm. (**B**) Hela cells were co-transfected with NS1-HA and Belcin-1-Flag plasmids for 48 h. Cells were lysed and the cell lysates were immunoprecipitated by anti-Flag Abs followed by immunoblotting using anti-HA and anti-Flag Abs. The whole cell lysate (WCL) was used as the protein control. (**C**) A549 cells were infected with DENV at MOI = 2 for 24 h. At 24 h post-infection, cells were fixed, permeabilized, and stained with anti-NS1 Abs (green), anti-Beclin-1 Abs (red) and DAPI (blue). Cells were mounted and observed by confocal microscopy. The square insets show higher magnification. Percentages of colocalized dots of NS1-Beclin-1 complex were indicated in the upper right corner. Scale bar: 10 μm. (**D**) Cells were lysed and the cell lysates were immunoprecipitated by anti-Beclin-1 Abs followed by immunoblotting using anti-NS1 and anti-Beclin-1 Abs. The whole cell lysate (WCL) was used as the protein control.

As shown in Figure 2, Beclin-1 is preserved in the early stage of DENV infection, and this may lead to a maintenance of autophagic activities for viral replication. Previous studies revealed that N-terminal arginylated Bip (R-BiP)/Beclin-1/p62 complex formation or cytosolic high-mobility group protein 1 (HMGB-1)/Beclin-1 interaction could prevent Beclin-1 cleavage [30,31]. Thus, we hypothesized that Beclin-1 may be protected from caspase-triggered degradation by interacting with viral factors in the early stage of infection. To elucidate which viral protein was responsible for protecting Beclin-1 from degradation, we introduced HA-tag DENV protein plasmids [42] and Beclin-1-Flag plasmids into Hela cells and performed confocal microscopy. We found colocalization of Beclin-1 with NS1; however, no colocalization was observed in cells with NS2B, NS3, NS4A and NS5 overexpression (Figure 4A). The interaction between ectopically expressed NS1 and Beclin-1 was further confirmed by a co-immunoprecipitation assay (Figure 4B). To determine this phenomenon during natural infection, the colocalization of NS1 and Beclin-1 was analyzed and observed by confocal microscopy in DENV-infected cells. About 35% of infected cells show positive dots in yellow (Figure 4C). This interaction was further confirmed by immunoprecipitation with anti-Beclin-1 Abs followed by immunoblotting analysis (Figure 4D). These data demonstrated an interaction between NS1 and Beclin-1 in DENV-infected cells.

**Figure 5:**
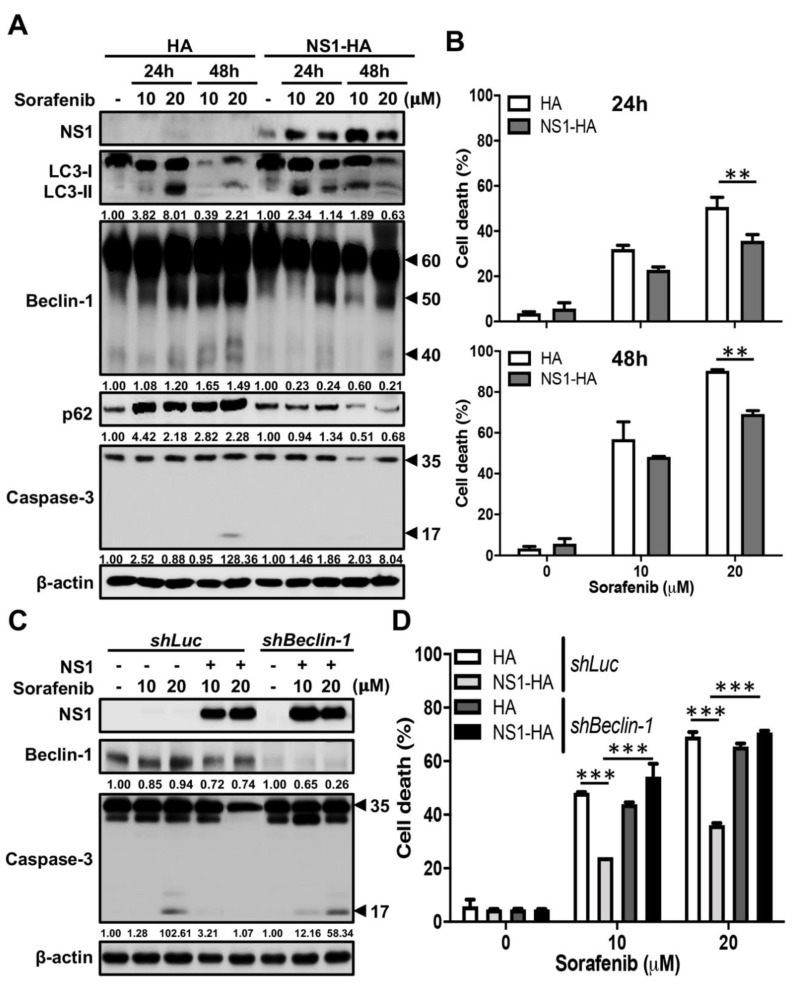
Ectopic expression of DENV NS1 attenuates sorafenib-induced cleavage of Beclin-1 and cell death. (**A**) A549 cells were transfected with a HA or NS1-HA plasmids and incubated with or without 10 or 20 μM sorafenib for 24 and 48 h. Western blot analysis showing representative images of NS1 levels, LC3-II accumulation, Beclin-1 levels (full-length: 60 kDa, cleaved form: around 50 and 40 kDa), p62 degradation as well as procaspase-3 (35 kDa) and cleaved caspase-3 (17 kDa) levels in A549 cells. β-actin is shown for loading control. The blots were quantified by using β-actin as a calibrated control. The relative ratios (numbers below the blots) of LC3-II, cleaved Beclin-1 (40 kDa), p62 or cleaved caspase-3 to sorafenib non-treated group were indicated. (**B**) The percentage of cell death was determined by propidium iodide staining at 24 (upper panel) and 48 (lower panel) hours, respectively. Results shown are means ± SD for triplicate cultures. Statistical significance was based on two-way ANOVA followed by Bonferroni’s multiple comparison test. ** *p* < 0.01. (**C**) A549 cells with Beclin-1 knockdown or Luc knockdown were transfected with HA or NS1-HA plasmids and treated with or without 10 or 20 μM sorafenib for 48 h. Western blot analysis showing representative images of NS1 and Beclin-1 levels as well as procaspase-3 (35 kDa) and cleaved caspase-3 (17 kDa) levels in A549 cells. β-actin is shown for loading control. The relative ratios (numbers below the The blots were quantified by using β-actin as a calibrated control. band) of Beclin-1 or cleaved caspase-3 to sorafenib non-treated group were indicated. (**D**) The percentage of cell death was determined by propidium iodide staining. Results shown are means ± SD for triplicate cultures. Statistical significance was based on two-way ANOVA followed by Tukey’s multiple comparison test. *** *p* < 0.001.

Next, we investigated whether NS1 is able to protect Beclin-1 from caspase cleavage to inhibit apoptosis and in turn to activate autophagy. We screened three apoptosis-induced drugs, including AR-12, cisplatin and sorafenib, by testing their activities to trigger Beclin-1 cleavage by Western blot analysis and apoptotic cell death by propidium iodide staining. Results showed that only sorafenib induced Beclin-1 cleavage, caspase-3 activation, and cell death in a dose- and time-dependent manner by Western blot analysis (Figure S3). Taking advantage of this sorafenib-induced cell death model, we examined whether the interaction of NS1 and Beclin-1 protects cells from apoptosis. We transfected NS1-expressing plasmids into A549 cells and then treated cells with sorafenib. Results showed that treatment with sorafenib induced LC3-II conversion, but also p62 accumulation along with Beclin-1 fragmentation, indicating an incomplete autophagic flux in control A549 cells by Western blot analysis (Figure 5A). However, ectopic expression of NS1 not only triggered LC-II conversion but also p62 degradation and reduced fragmentation of Beclin-1 under sorafenib-treatment conditions (Figure 5A). In addition, sorafenib-induced caspase-3 cleavage (Figure 5A) and cell death (24 h post-treatment: 50.7 ± 7.44% versus 35.73 ± 4.76%, about 0.70-fold change, *p* < 0.01; 48 h post-treatment: 90.37 ± 1.00% versus 69.17± 3.02%, about 0.77-fold change, *p* < 0.01) (Figure 5B) were both significantly decreased in NS1-expressing cells. These data indicate that NS1 is able to protect Beclin-1 from cleavage and hence maintains autophagy to anti-apoptosis. Notably, expression of NS3 could not inhibit sorafenib-triggered caspase-3 activation and Beclin-1 fragmentation (Figure S4A) as well as cell death (10 mM sorafenib treatment: 56.83 ± 14.79% versus 70.67 ± 1.15%, about 1.24-fold change, *p* = 0.23; 20 mM sorafenib treatment: 82.17 ± 14.89% versus 81.03 ± 0.91%, about 0.99-fold change, *p* > 0.99) (Figure S4B), indicating the essential role of NS1 in this Beclin-1-mediated anti-apoptotic activity.

To further verify the possible roles of Beclin-1 involvement in the anti-apoptotic effect of NS1, the shBeclin-1 and shLuc cells were transfected with NS1-containing plasmids and treated with sorafenib. Results showed that expression of NS1 reduced sorafenib-induced cleavage of caspase-3 (Figure 5C) and cell death (Figure 5D) (10 mM sorafenib treatment: 48.17 ± 0.45% versus 24.12 ± 0.02%, about 0.50-fold change, *p* < 0.001; 20 mM sorafenib treatment: 69.17 ± 3.02% versus 36.16± 1.18%, about 0.52-fold change, *p* < 0.001) in shLuc cells. In contrast to control shLuc cells, this phenomenon did not occur in Beclin-1 knockdown cells (Figure 5C,D) (10 mM sorafenib treatment: 43.93 ± 1.25% versus 54.19 ± 8.38%, about 1.23-fold change, *p* < 0.01; 20 mM sorafenib treatment: 65.47 ± 1.96% versus 70.67± 1.15%, about 1.08-fold change, *p* = 0.17). To conclude, our data suggest that NS1, but not NS3, promotes autophagy by attenuating Beclin-1 degradation.

## 3. Discussion

**Figure 6:**
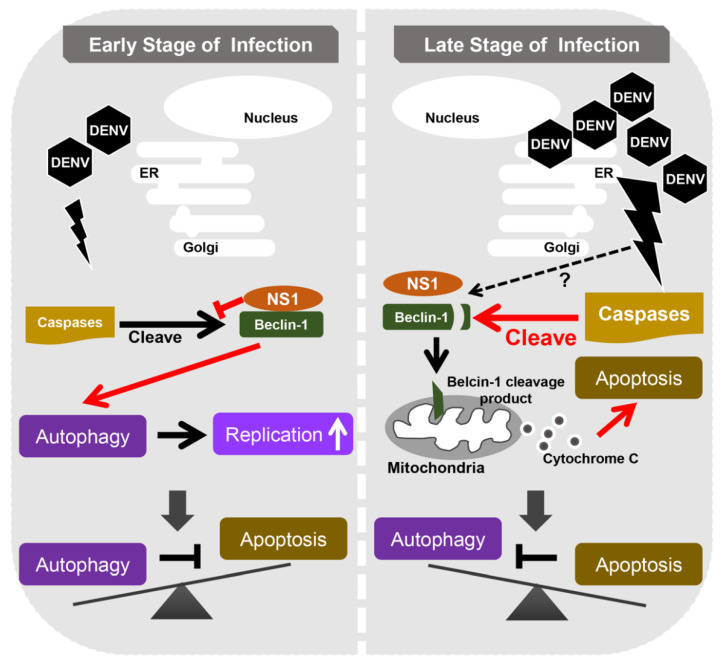
The proposed schematic model of Beclin-1 stability in regulating autophagy and apoptosis during DENV replication. DENV infection activates both autophagy and apoptosis. In the early stage of infection, DENV NS1 interacts with Beclin-1 to interfere with caspase-mediated Beclin-1 cleavage and hence facilitates autophagy to inhibit early apoptotic cell death, and therefore helps DENV replication. However, in the late stage of infection (after 48 h post infection), higher levels of activated caspases cleave Beclin-1 into fragments. This may lead to autophagy attenuation and cause severe apoptotic cell death.

In this study, we highlighted the important role of Beclin-1 in the DENV life cycle and found that Beclin-1 is involved in the interplay of autophagy and apoptosis to control viral replication. Moreover, we provided a potential mechanism for how DENV-induced autophagy suppresses early apoptosis. The interaction of DENV NS1 and Beclin-1 could block the caspase-mediated cleavage of Beclin-1 to maintain autophagy, which then protects infected cells from early apoptotic death (Figure 6).

As we know, autophagy and apoptosis control the homeostasis within cells; therefore, many stimuli or stress pathways sequentially elicit autophagy and apoptosis within the same cell. In general, autophagy blocks the induction of apoptosis and helps cells to adjust to environmental stress; however, accumulated external signals may in turn activate apoptosis-associated molecules and shut off the autophagic process. Both of these processes are under the control of multiple common upstream signals, and usually the cross-regulation between them is of an inhibitory manner [21]. In this study, we revealed that the stability of Beclin-1, through its interaction with NS1, plays a crucial role in regulating DENV-induced autophagy and apoptosis. However, our results showed that even the expression levels of NS1 were increased in the late stage of DENV infection, and Beclin-1 was still cleaved by caspases (Figure 3A). The detailed mechanisms are still not clear, but they might be associated with the activity of caspases induced by DENV infection. Previous studies have demonstrated that several caspases, including caspase-1, -3, -4, -6, -7, -8 and -9, were involved in DENV-induced apoptosis. Of note, the activities of these caspases were increased in a time-dependent manner [43,44,45,46]. In our study, we explored the enhanced activities of caspase-3 (Figure 1, Figure 2 and Figure 3) as well as caspase-8 and -9 (data not shown) in the late stage of DENV infection. A previous study has demonstrated that the cleaved Beclin-1 enhances pro-apoptotic effects by inducing cytochrome C release from mitochondria [33]. Therefore, it is suggested that DENV infection causes a low level of activated caspases in the early infection stage, which still allows DENV NS1 to protect Beclin-1 from caspase-mediated cleavage. However, if the death signal induced by DENV reaches the stress threshold, the activated caspases might interfere with NS1 protection and hence trigger cell death by cleaving Beclin-1.

Caspases are proteases and serve as molecular switches between autophagy and apoptosis by cleaving various autophagy associated proteins. The cleaved fragments of autophagy-associated proteins act as apoptosis inducers rather than autophagy inducers [33]. Beclin-1 was cleaved by multiple caspases and turned to several fragments with different molecular weights. For example, IL-3 depletion in Ba/F3 cells enhanced the caspase-mediated cleavage of Beclin-1 to generate fragments with 50 kDa, 37 kDa and 35 kDa [33]. Results from an in vitro caspase cleavage assay indicated that Beclin-1 was directly cleaved into fragments with 50 kDa and 42–43 kDa by caspase-3 but not caspase-8. Meanwhile, Beclin-1 was cleaved into only one fragment with 50 kDa by caspase-9 and only one fragment with 42–43 kDa by caspase-6 [47]. Another study showed that Beclin-1 could be strongly cleaved by active caspase-8, weakly by active caspase-3, but not by caspase-9, and turned into fragments of around 40 and 35 kDa. In addition, both the aspartic acids 133 and 146 on Beclin-1 could be the potential caspase recognized sites [36]. In our study, we found different sizes of Beclin-1 fragments, including the clear one or two bands around 50 and 40 kDa (Figure 2A), and the other diffused smear pattern between 50 and 40 kDa (Figure 3A). This suggests that the cleavage of Beclin-1 during DENV infection might be mediated by caspase-3, -6, -8 or -9. However, further investigation is required to understand their roles in contributing to Beclin-1 cleavage and the consequences of Beclin-1 fragments during DENV infection.

Autophagy is initiated by isolation membrane (also called a phagophore) formation. The origin of the isolation membrane can be generated from ER, Golgi apparatus, mitochondria, plasma membrane, or their contact sites such as ER–mitochondria contact sites [9]. Through a series of reactions, this isolation membrane is subsequently processed including nucleation, elongation, autophagosome maturation, and fusion between the autophagosome and lysosome for degradation. Beclin-1 serves as a scaffold platform for multiprotein assembly during autophagy initiation and nucleation. Hence, Beclin-1 is located in many organelles including ER, Golgi apparatus and mitochondria [29,36]. The DENV genome also translates the viral proteins in ER. After viral genome translation, DENV NS1 becomes a membrane-associated homodimer and participates in a viral replication complex [48,49]. A previous study showed that intracellular NS1 predominantly localizes in the perinuclear regions of DENV-infected cells [50]. The other study revealed that NS1 not only localizes in viral RNA replication sites, but also in ER during DENV infection [51]. In our study, we also found the co-localization of NS1 and Beclin-1 in the perinuclear regions of DENV-infected cells (Figure 4C) as well as direct interaction between these two molecules (Figure 4D). Moreover, according to Figure S5, NS1 is expressed between the ER marker (sec61β) and mitochondria marker (Tom20). This suggests that NS1 may express at ER–mitochondria contact sites where Beclin-1 is recruited when autophagy is induced [52]. Although the distribution of NS1-Beclin-1 complex in DENV-infected cells still need to be further investigated, we suggest that Beclin-1 may interact with NS1 mainly in the viral replication complex within ER to induce autophagy and help the DENV replication. Confronted with a lack of Beclin-1, DENV cannot obtain enough ATP generated from autophagy-dependent processing of lipid droplets and triglycerides, and results in severe cell death to inhibit the viral replication [53,54]. Our results also showed that Beclin-1 plays a positive role for DENV replication (Figure 2D). However, the detailed mechanisms need to be further investigated and verified in a DENV-infected animal model.

Beclin-1 contains three conserved structural domains, the Bcl-2 family binding BH3 domain (a.a. 105–130), CCD (a.a. 175–265) and ECD (a.a. 248–337); all three domains enable Beclin-1 to interact with multiple proteins for modulating autophagy [9,29]. The cleavage products of N-terminal (a.a. 1–133) and C-terminal (a.a. 150–450) Beclin-1 fail to induce autophagy; however, the full-length Beclin-1 clearly induces an autophagic response and remains in cytoplasmic distribution. The *N*-terminal fragments are accumulated near the nucleus while the C-terminal fragments are targeted to mitochondria to promote apoptosis [33,35]. On the other hand, the formation of R-BiP/Beclin-1/p62 complex can inhibit caspase-mediated cleavage, which triggers autophagy to protect cells from apoptosis [31]. Cytosolic HMGB1 regulates apoptosis by protecting Beclin-1 and ATG5 from calpain-mediated cleavage during inflammation [30]. Consistent with these previous studies, our results demonstrated that the interaction of DENV NS1 and Beclin-1 attenuated Beclin-1 degradation and promotes autophagy to inhibit apoptotic cell death (Figure 4 and Figure 5). However, DENV infection still leads to Beclin-1 degradation at a later phase of the replication cycle (Figure 2A). This suggests that the cleavage of Beclin-1 at latter time might be mediated by several caspases together or the interaction is transient to allow further cleavage. Hence, the understanding of crucial domains within DENV NS1 interacting with Beclin-1 and the dynamic change of this complex in DENV infection remain to be elucidated. This is the first study to suggest the potential role of Beclin-1 in the interplay of the DENV induced autophagy and apoptosis. We also revealed that DENV NS1 contributes to enhancing viral replication through maintaining autophagy at early infection. The possible implications of the findings in this study are valuable for developing new strategies of antiviral drugs. In particular, targeting the interruption of NS1 and Beclin-1 interaction will be helpful to prevent further viral multiplication.

## 4. Materials and Methods

### 4.1. Cell Cultures

C6/36 cells were cultured in Roswell Park Memorial Institute medium (RPMI) 1640 (Invitrogen, Carlsbad, CA, USA) containing antibiotics, 1% penicillin/streptomycin (P/S, Invitrogen), and 10% fetal bovine serum (FBS, HyClone, South Logan, UT, USA) at 28 °C in 5% CO_2_. Baby hamster kidney cells (BHK-21) cells were cultured in RPMI 1640 containing 5% FBS at 37 °C in 5% CO_2_. Human lung carcinoma epithelial cells A549 and cervical carcinoma epithelial cells Hela were both cultured in Dulbecco’s modified Eagle’s medium (DMEM, Invitrogen) containing antibiotics and 10% FBS at 37 °C in 5% CO_2_.

### 4.2. Virus Cultures

DENV2 (strain PL046) was propagated in C6/36 cells. C6/36 monolayers were infected with DENV at a multiplicity of infection (MOI) of 0.01 and further incubated with DMEM containing 2% FBS at 28 °C for 5 days. To collect the virus, the culture medium was centrifuged at 400× *g* for 10 min at 4 °C, and the virus supernatant was collected as well as stored at −70 °C until use in experiments. Plaque assay was applied to determine viral titer in BHK-21 cells.

For plaque assay, BHK-21 cells were seeded in 12-well culture plates (8 *×* 10^4^ cells/well) and then cultured in RPMI medium containing 5% FBS overnight. On the second day, RPMI medium was removed and then a serial diluted virus solution in serum free RPMI was added into the wells for 2 h incubation. Later, the virus solution was replaced with fresh DMEM containing 2% FBS and 0.5% methyl cellulose (Sigma-Aldrich, St. Louis, MO, USA). Seven days after infection, the cells were washed by PBS, fixed, and stained with crystal violet solution (1% crystal violet, 0.64% NaCl, and 2% formalin) for 1 day. Finally, the crystal violet solution in plates was washed, and then plates were air-dried to count the number of plaque-forming units (PFU).

### 4.3. DENV Infection

A549 cells were seeded in 6-well plates (2 × 10^5^ cells/well) or 12-well plates (1 × 10^5^ cells/well) for one day before infection. A549 cells were inoculated with DENV (MOI = 2) for 2 h at 37 °C (shaking the plate every 30 min). After 2 h post-infection, cells were washed with PBS and further incubated before harvesting.

### 4.4. Western Blotting and Immunoprecipitation

For whole cell extracts, the cells were lysed with a buffer containing 20 mM Tris-HCl pH 7.5, 150 mM NaCl, 1 mM Na_2_EDTA, 1 mM EDTA, 1% Triton, 2.5 mM sodium pyrophosphate, 1 mM β-glycerophosphate, 1 mM Na_3_VO_4_, 1 μg/mL leupeptin) on ice for 10 min. After being freeze-thawed once, cell lysates were centrifuged at 12,000 rpm at 4 °C for 20 min. The supernatant was collected and boiled in sample buffer for 15 min. The whole cell extracts were resolved on SDS-PAGE and electro-transferred onto polyvinylidene difluoride membranes (Millipore, Billerica, MA, USA). After blocking with 5% nonfat milk, the blots were incubated with rabbit Abs against LC3 (MBL International Corporation, Nagoya, Japan), Abs against p62 (MBL International Corporation) and Abs against caspase-3 (Cell Signaling Technology, Beverly, MA, USA), or mouse Abs against Beclin-1 (Millipore) and Abs against DENV2 NS1 (clone 33D2, obtained from Trai-Ming Yeh) overnight at 4 °C. Mouse Abs specific for β-actin (Sigma-Aldrich) were used for internal controls. Finally, blots were hybridized with horseradish peroxidase-conjugated goat anti-rabbit or anti-mouse IgG (Cell Signaling Technology) and developed using western lightning plus ECL enhanced Luminol Reagent Plus (PerkinElmer, Boston, MA, USA).

At 24 h post-infection with DENV (MOI = 2), the cell lysates were immunoprecipitated by anti-Beclin-1 Abs followed by immunoblotting using anti-NS1 and anti-Beclin-1 Abs. The whole cell lysate (WCL) was used as the protein control.

### 4.5. Lentiviral-Based RNAi Gene Knockdown

Beclin-1 was silenced in A549 cells by stably expressing the lentiviral-based short hairpin RNA (shRNA) targeting the human Beclin-1. The Beclin-1 shRNA sequence is 5′-CCCGTGGAATGGAATGAGATT-3′ and the negative control Luciferase shRNA sequence is 5′-ATCACAGAATCGCGTATGCA-3′. 293T/17 cells were used to produce lentiviruses by co-transfection of the DNA mixture (pCMV-dR8.91, pMD.G and shRNA from National RNAi Core Facility, Taiwan) using GeneJammer Transfection Reagent (Agilent Technologies Stevens Creek Blvd Santa Clara, CA, USA). Cells were infected by lentiviruses with an appropriate MOI in growth medium supplemented with polybrene (Sigma-Aldrich). Cells were then changed to puromycin (Sigma-Aldrich) containing medium after infection for 48 h, and stably expressed shRNA cells were selected. The silencing efficacy of protein was determined by Western blotting.

### 4.6. Cell Death Determination

For the detection of cell death rate, 2 × 10^5^ or 1 × 10^5^ cells were seeded into 6 or 12 well plates, respectively. After DENV infection or treating with inhibitors, the washed A549 cells were stained with propidium iodide staining buffer (2% FBS, 0.1% NaN_3_, 10 μg/mL propidium iodide in 500 mL PBS) on ice for 30 min. The cell death was detected and analyzed using flow cytometry (FACScan™; BD Biosciences, San Jose, CA, USA).

### 4.7. Immunofluorescence Assay (IFA)

For immunofluorescence staining, cells were grown on glass slides and infected with DENV as described above. For colocalization assay, cells were fixed with 4% paraformaldehyde for 10 min at room temperature. After washing, cells were stained with Abs against DENV NS1 and Abs against Beclin-1 (Cell Signaling Technology) at 4 °C overnight followed by Alexa 488-conjugated goat anti-mouse IgG and Alexa-594-conjugated goat anti-rabbit IgG (Invitrogen) for 2 h at room temperature. The nuclei of cells were stained with 4’, 6-diamidino-2-phenylindole (DAPI; Calbiochem, San Diego, CA, USA). Fluorescence image studies were performed using a laser confocal microscope (Olympus FV-1000).

### 4.8. Plasmid Transfection

For cell transfection, 2 × 10^5^ or 1 × 10^5^ cells were seeded into 6 or 12 well plates, respectively. Two μl Lipofectamine R3000 (Invitrogen) plus 1 μg of HA, NS1-HA, NS2B-HA, NS3-HA, NS4A-HA, NS5-HA (kindly provided by Chia-Yi Yu) or Beclin-1-Flag (kindly provided by Tamotsu Yoshimori, Department of Genetics, Graduate School of Medicine, Osaka University) plasmids were diluted in serum-free DMEM and incubated for 20 min at room temperature. Mixtures were added to cells and incubated for 48 h at 37 °C.

### 4.9. Statistical Analysis

Data are expressed as the mean ± SD of triplicate cultures. Multiple intergroup comparisons were assessed by two-way ANOVA when appropriate, followed by post-hoc tests (mostly Bonferroni’s test and Tukey’s test) with GraphPad Prism version 8.0. Statistical significance was set at *p* < 0.05.

## 5. Conclusions

In conclusion, Beclin-1 plays important roles in convergence between autophagy and apoptosis during DENV infection. In the early stage of infection, the viral protein NS1 prevents Beclin-1 from cleavage to maintain autophagy, which inhibits early apoptosis and increases viral replication. With the accumulation of cellular stresses, the activation of caspases leads to Beclin-1 cleavage and hence enhances cell apoptosis in the late stage of DENV infection.

## Supplementary Materials

Supplementary materials can be found at https://www.mdpi.com/1422-0067/21/24/9702/s1.

## Abbreviations

3-MA	3-Methyladenine
ATG	Autophagy related genes
BH3	Bcl-2 homology 3
Bip	Binding immunoglobulin protein
CCD	Coiled-coil domain
DENV	Dengue virus
ECD	Evolutionarily conserved domain
ER	Endoplasmic reticulum
GRP78	78-kDa glucose-regulated protein
HMGB-1	High-mobility group protein 1
IRE1α	inositol-requiring protein-1α
JNK	c-Jun N-terminal kinase
kDa	kilo-Dalton
LC3	Microtubule-associated protein 1 light chain 3 alpha
Ab	Antibody
MOI	Multiplicity of infection
NS	Nonstructural protein
p62	Sequestosome 1; SQSTM1
PI3K	phosphatidylinositol 3 kinase
R-Bip	arginylated Bip
