# Supplementary material for: Dengue Nonstructural Protein 1 Maintains Autophagy through Retarding Caspase-Mediated Cleavage of Beclin-1
Source: Int J Mol Sci. 2020 Dec 19;21(24):9702. doi: 10.3390/ijms21249702 (PMC7766445; doi:10.3390/ijms21249702)
Supplement: Supplementary file 1 [file ijms-21-09702-s001.pdf]

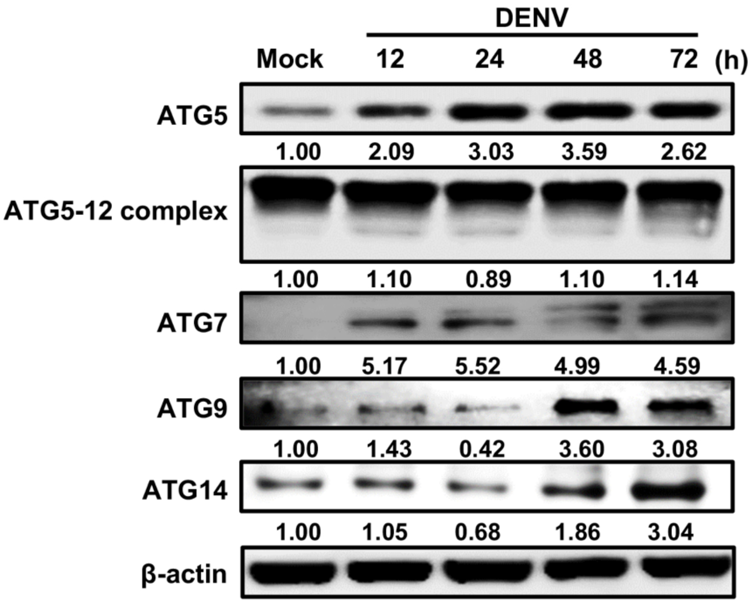

Figure S1. The expression level of ATG proteins in in DENV-infected cells. A549 cells were infected with DENV at MOI=2 for indicated time. Western blot analyzed showing representative images of ATG5, ATG5-12 complex, ATG7 and ATG14 levels in mock and DENV-infected cells. β –actin was shown for loading control. The blots were quantified by using β-actin as a calibrated control. The relative ratios (numbers below the blots) of protein expression to the mock were indicated.

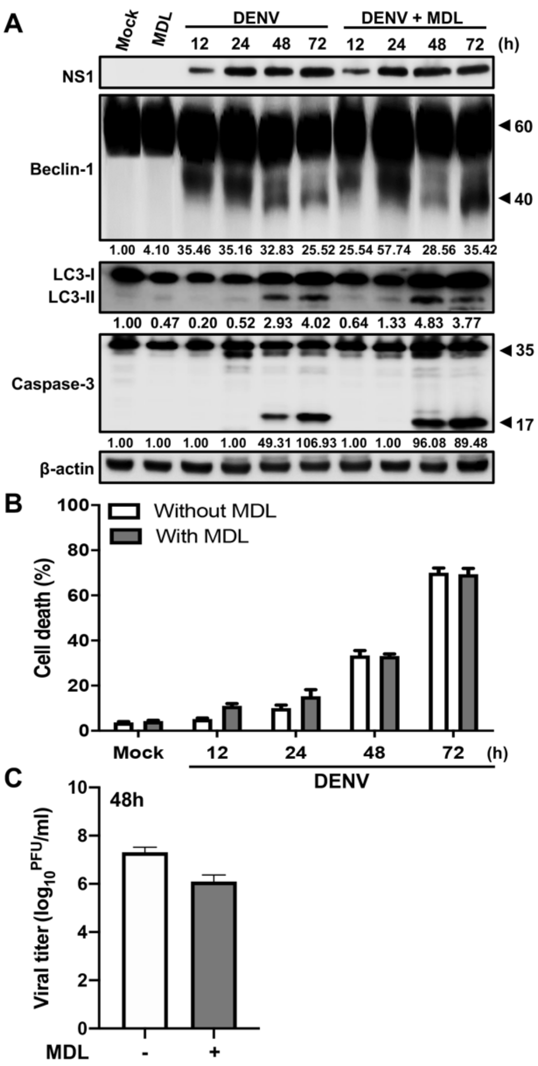

Figure S2. The cleavage of Beclin-1, cell death and virus replication are not mediated by calpain in the DENV-infected cells. A549 cells were pretreated with or without 50 μM MDL28710 and then infected with DENV at MOI=2 for the indicated time. **(A)** Western blot analyzed showing representative images of NS1 levels, Beclin-1 levels (full-length: 60 kDa, cleaved form: around 50 and 40 kDa), LC3-II accumulation, as well as procaspase-3 (35 kDa) and cleaved caspase-3 (17 kDa) levels in A549 cells. β -actin was shown for loading control. The blots were quantified by using β-actin as a calibrated control. The relative ratios (numbers below the blots) of LC3-II, cleaved Beclin-1 (40 kDa) or cleaved caspase-3 to the mock were indicated. **(B)** The percentage of cell death was determined by propidium iodide staining. Results shown are means ± SD for triplicate cultures. Statistical significance was based on two-way ANOVA followed by Bonferroni's multiple comparison test. **(C)** The viral titers were determined by plaque assay. Results shown are means ± SD for triplicate cultures. Statistical analysis was performed using a two-tailed Student t-test. All the data are representative of independent experiments.

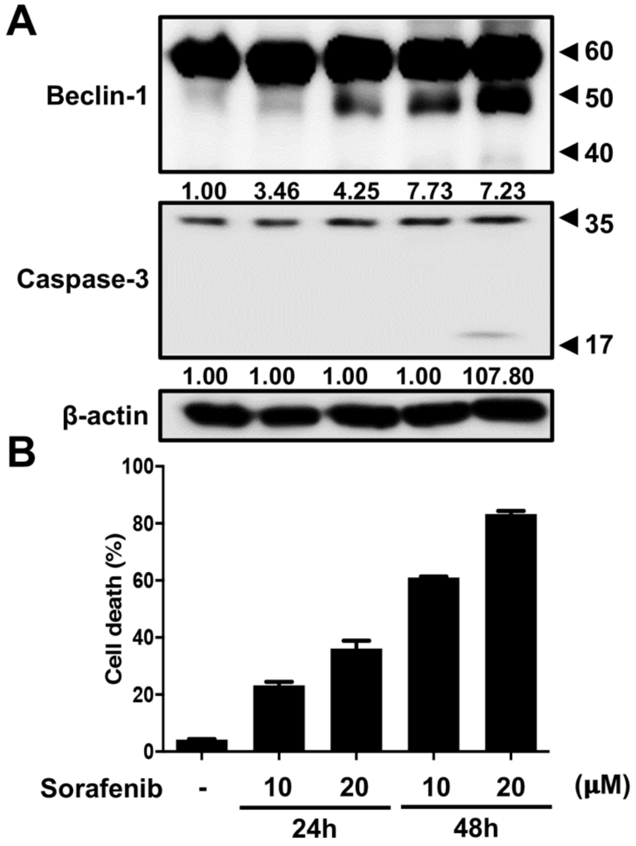

Figure S3. Sorafenib induces Beclin-1 cleavage and cell death. A549 cells were treated with 10 or 20 μM sorafenib for 24 and 48 hours. **(A)** Western blot analyzed showing representative images of Beclin-1 levels (full-length: 60 kDa, cleaved form: around 50 and 40 kDa), as well as procaspase-3 (35 kDa) and cleaved caspase-3 (17 kDa) levels in A549 cells. β -actin was shown for loading control. The blots were quantified by using β-actin as a calibrated control. The relative ratios (numbers below the blots) of cleaved Beclin-1 (40 kDa) or cleaved caspase-3 to sorafenib non-treated group were indicated. **(B)** The percentage of cell death was determined by propidium iodide staining.

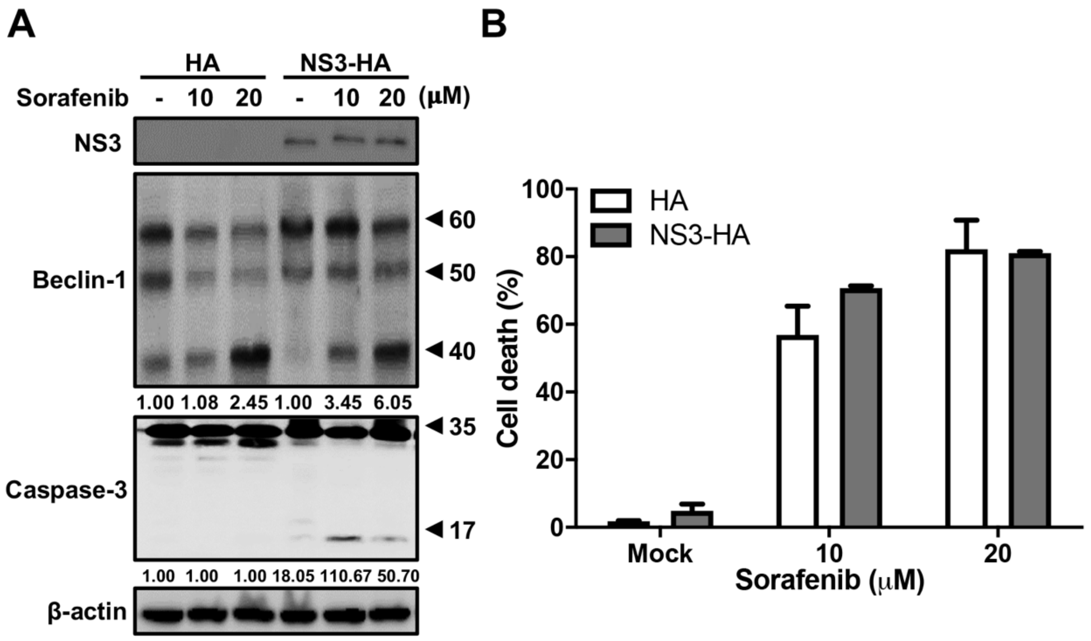

Figure S4. Expression of DENV NS3 has no effects on sorafenib-induced cleavage of Beclin-1 and cell death. **(A)** A549 cells were transfected with a HA or NS3-HA plasmid and incubated with or without 10 or 20 μM sorafenib for 48 hours. Western blot analyzed showing representative images of NS3 levels, LC3-II accumulation, Beclin-1 levels (full-length: 60 kDa, cleaved form: around 50 and 40 kDa) as well as procaspase-3 (35 kDa) and cleaved caspase-3 (17 kDa) levels in A549 cells. β –actin was shown for loading control. The blots were quantified by using β-actin as a calibrated control. The relative ratios (numbers below the blots) of cleaved Beclin-1 (40 kDa) or cleaved caspase-3 to the sorafenib non-treated group were indicated. **(B)** The percentage of cell death was determined by propidium iodide staining. Results shown are means ± SD for triplicate cultures. Statistical significance was based on two-way ANOVA followed by Bonferroni's multiple comparison test.

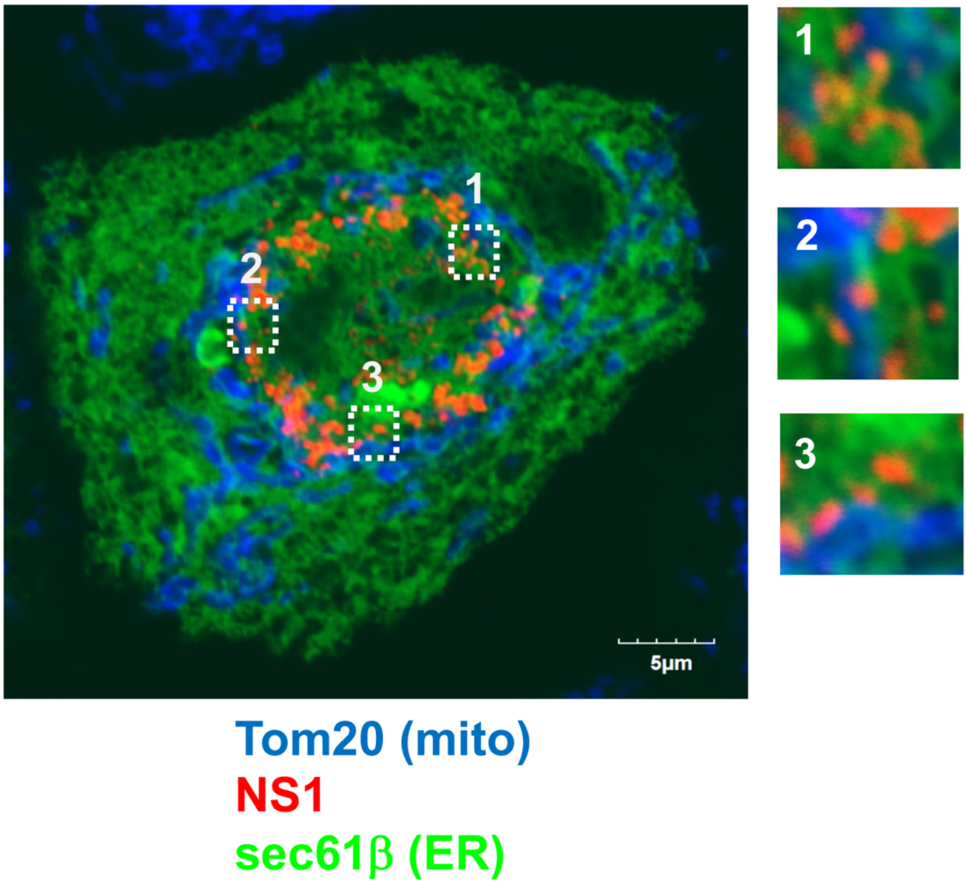

Figure S5. DENV NS1 is expressed at ER-mitochondria contact sites. Hela cells were co-transfected with NS1-HA and sec61β-GFP plasmids for 48 hours. At 48 hours post-transfection, cells were fixed, permeabilized, and stained with anti-Tom20 Abs (blue) and anti-HA Abs (red). Cells were mounted and observed by confocal microscopy. The square insets show higher magnification.
